# Conducting a sensitive, constructive and ethical peer review

**DOI:** 10.1002/nop2.944

**Published:** 2021-05-21

**Authors:** Daniel Bressington, David R. Thompson, Martin Jones, Richard Gray

**Affiliations:** ^1^ College of Nursing & Midwifery Charles Darwin University Casuarina NT Australia; ^2^ School of Nursing and Midwifery Queen's University Belfast Belfast UK; ^3^ Department of Psychiatry University of Melbourne Melbourne Vic Australia; ^4^ Department of Rural Health University of South Australia Whyalla SA Australia; ^5^ La Trobe University Melbourne Vic Australia

We were inspired to write this editorial by our experiences of editing/reviewing for several journals and an article was written by Wiley's Regional Manager of Peer Review imploring editors to “Do to Others as You Would Have Them Do to You” in order to foster academic kindness (Willis, 2020). Clearly, editors have a responsibility to ensure that reviewers' reports are not released to authors when there are serious problems or if they are obviously disparaging or, indeed, even libellous. As editors, we can rescind reviews or ask reviewers to amend their comments. But when there are more subtle insensitivities (such as blunt comments or a lack of politeness) it is often difficult to address, and we are aware of the need not to delay the review process by sending the reviews back unless it is essential. Hence, we would like to share some of our thoughts on the peer review process with the aim of encouraging authors and reviewers and to make the peer review process a constructive and enriching experience on both sides. We also hope to foster academic kindness by encouraging constructive yet sensitive reviews that help to maintain the mental well‐being of authors.

Peer review is a central (and arguably the most important) aspect of the scientific publishing process (Kelly et al., 2014). Editors rely on peer reviewers to provide an expert assessment of the value, quality and accuracy of a submitted manuscript to inform their decision about whether to publish or reject. Without peer reviews, it is impossible to maintain the checks and balances necessary to ensure articles meet the required standard. Researchers who publish their work have an obligation to reciprocate to engage with the peer review process as a reviewer. It has been argued that for the scientific peer review machine to work researchers need to complete at least two to three reviews for every one paper they submitted, which may rise to up to 15 reviews (shared across a research team) when they publish a manuscript in a journal with an 80% rejection rate (Fernandez‐Llimos et al., 2020). Given that so little academic credit is awarded to reviewers for completing timely reviews it is perhaps not surprising that it can be challenging to get colleagues to complete timely high‐quality reviews.

The Committee On Publication Ethics (COPE, 2013) provide clear ethical guidelines for peer reviewers. Some of the basic principles include respecting confidentiality, having the required expertise to conduct the review, not using the process to gain an academic advantage (or to disadvantage others), providing accurate information, being objective and constructive and appreciating the reciprocal nature of peer review. As editors, we delight when we receive balanced, ethical, constructive and well‐articulated peer reviews, but our hearts often sink when reviews are critical, unconstructive or dismissive. If we feel this way as editors, you can imagine (and have likely experienced) the affect‐shifting impact of getting an insensitive negative peer review when you open the long‐awaited email with a subject title along the lines of: “Decision for manuscript number 1234X…”.

Although most authors, editors and reviewers must recognise that our academic work needs to be questioned, critiqued and reinterpreted in order to further science, ultimately, we are human beings that are emotionally bound to our research (Cunningham et al., 2021). This emotional connection means we are likely to take criticism personally. Therefore, the potential damage to authors' mental health and well‐being when receiving an unfair and insensitive peer review should not be underestimated. Particularly as most researchers and higher degree (HD) students are depending on getting their work published to progress their careers. It is increasingly apparent that the mental health of academics/HD students is particularly fragile in the highly competitive world of academia (Bira et al., 2019; Lashuel, 2020). Relatively few studies have examined the mental health of academics; some indication is provided by Schindler et al. (2006) who concluded that 20% of over 1900 full‐time academics at four medical schools reported significant levels of depressive symptoms and that levels of depression were commonly related to work‐related strain. The mental health status of HD students is more comprehensively studied, as illustrated by an international study highlighting that 39% of 2,279 graduate students from across 26 countries reported moderate to severe depression (Evans et al. 2018). Given these potential mental health related challenges, we hope that reviewers will be aware of and sensitive to the need for academic kindness.

We hope we have encouraged some reflection of the need for sensitive peer review. Having thought about this issue ourselves these are some of our do's and don'ts:

- Do use polite language. Please and thank you go a long way. So please, for example, consider writing “I wonder if the author could please check a make sure that they have followed the appropriate reporting guidelines…”
- Don't be rude or condescending in tone. Don't write “this work would be poor if it had come from an undergraduate from a lesser University, clearly this is not publishable.”
- Do assume the best, be humble and appreciate what you may have misunderstood. If you detect a problem, assume that you may be wrong. This is equally important when you think you may have identified potential research misconduct, so give the authors an opportunity to clarify where appropriate.
- Do provide clear and precise feedback about what the authors need to change (and why and how). Consider writing “I'd be grateful if the authors could provide precise details about how they randomised (allocation concealment, sequence generation) in their study, I'd suggest looking at the following guidance for further information.”
- Don't make general sweeping statements, such as “mixed methods research is simply a way of dressing up a substandard study as something interesting, I do not have time for it”.
- Do outline the strengths of the manuscript, even if you think it is inherently flawed and should not be published. “I congratulate the authors on the effort that they have put into this manuscript. Unfortunately, I think there are some important methodological issues that will need to be addressed, for example…”.
- Do comment if poor grammar detracts from clarity. But, don't focus too much on individual typos or make insensitive comments about authors not being native English speakers. For example, it might be appropriate to write “I note that you are inconsistent in your use of past and present tenses, as you are reporting the results of a study that has been completed you should use the past tense for example we randomised…, participants were assessed by a researcher blind to…”
- One bugbear of ours is self‐citation in peer review. Don't use the process as an opportunity to put pressure on the authors to cite your (or your mates') work in the manuscript. Interestingly, Thombs et al. (2015) reported that 29% (122/428) of citations recommended for inclusion in manuscripts were peer reviewer self‐citations and in 21% of these no rationale was provided. Unfortunately, anecdotal evidence suggests this appears to be an ongoing issue‐ a recent review received by one of us suggested no less than 10 self‐citations, many of which were opinion‐based papers, and few were supported with a rationale. Of course, in this instance, the review was rescinded. So definitely do not write in a review, “the authors have omitted a number of important citations, MINE!! Make sure you include these in your revised manuscript or I will reject your paper.” Do provide a clear rationale and declare to the editor (in the confidential comments if it is a double‐blind peer review) if you have recommended any self‐citations and why these are necessary for the absence of any alternatives.
- Finally, before submitting read the review back and imagine how you would feel if you received the review you have just written.

We should be mindful and respectful that authors have spent a good deal of time and effort thinking about, preparing and submitting their paper. The least we can do as editors and peer reviewers are to acknowledge this regardless of whether the paper is accepted or rejected. It is our academic and professional duty to support authors – many of whom are submitting their first paper – who will invariably be grateful, learn from and improve their writing and publishing.

It is also worth acknowledging that peer reviewers undertake the task on top of often heavy workloads and with no remuneration. They may do it for a variety of reasons, including gaining peer recognition, keeping abreast of developments in their field, bolstering their CVs and maybe even aiding career progression. We encourage authors to make use of Publons (https://publons.com/about/home/) to track their peer reviews and encourage colleagues in academic leadership roles to recognise peer review and a performance metric. Reviewers are vital to the publishing enterprise and they serve an important role for the journal, the profession and the individual. But, as with all roles, there is always room for improvement.

## CONFLICT OF INTEREST

Daniel Bressington is Associate Editor for Nursing Open and Frontiers Public Mental Health. David R Thompson is Co‐Editor for the European Journal of Cardiovascular Nursing. Martin Jones is Associate Editor for the Australian Journal of Rural Health. Richard Gray is Editor in Chief for Nursing Reports.

## AUTHOR CONTRIBUTIONS

All authors have contributed equally, meet at least one of the ICMJE criteria for authorship (http://www.icmje.org/recommendations/) and have approved the final version.

## ETHICAL APPROVAL

Ethical approval is not for required this editorial.

## Data Availability

Data sharing not applicable – no new data generated.
